# Do you see what I hear? Vantage point preference and visual dominance in a time-space synaesthete

**DOI:** 10.3389/fpsyg.2013.00695

**Published:** 2013-10-16

**Authors:** Michelle Jarick, Mark T. Stewart, Daniel Smilek, Michael J. Dixon

**Affiliations:** ^1^Neurocognition of Attention and Perception Lab, Department of Psychology, MacEwan UniversityEdmonton, AB, Canada; ^2^Department of Psychology, Willamette UniversitySalem, OR, USA; ^3^Department of Psychology, University of WaterlooWaterloo, ON, Canada

**Keywords:** synaesthesia, spatial-cueing, spatial perception, attention, mental vantage points, orienting, reification

## Abstract

Time-space synaesthetes “see” time units organized in a spatial form. While the structure might be invariant for most synaesthetes, the perspective by which some view their calendar is somewhat flexible. One well-studied synaesthete L adopts different viewpoints for months seen vs. heard. Interestingly, L claims to prefer her auditory perspective, even though the month names are represented visually upside down. To verify this, we used a spatial-cueing task that included audiovisual month cues. These cues were either congruent with L's preferred “auditory” viewpoint (auditory-only and auditory + month inverted) or incongruent (upright visual-only and auditory + month upright). Our prediction was that L would show enhanced cueing effects (larger response time difference between valid and invalid targets) following the audiovisual congruent cues since both elicit the “preferred” auditory perspective. Also, when faced with conflicting cues, we predicted L would choose the preferred auditory perspective over the visual perspective. As we expected, L did show enhanced cueing effects following the audiovisual congruent cues that corresponded with her preferred auditory perspective, but that the visual perspective dominated when L was faced with both viewpoints simultaneously. The results are discussed with relation to the reification hypothesis of sequence space synaesthesia (Eagleman, 2009).

## Introduction

Synaesthesia is a fascinating phenomenon whereby ordinary sensory information perceived in one modality elicits a second extraordinary sensory experience in the same or different modality. The types of associations most studied include experiencing colors for letters and numbers (e.g., Dixon et al., 2000), specific tastes for words (e.g., Simner and Ward, 2006), and colors evoked by different musical tones (e.g., Cytowic and Eagleman, 2009). Here we examined an individual (L) who *sees time in space*, meaning that years, months, days, and hours elicit highly specific spatial locations that surround her. This type of association is encased under the larger umbrella term of sequence-space synaesthesia (SSS; Eagleman, 2009).

An intriguing theory of SSS is that the spatial forms are the objectification of overlearned sequences (the *reification hypothesis*; Eagleman, 2009). In other words, the spatial forms that synaesthetes report may be closer to the experience of real “objects” than mere figments of visualization/imagination. In fact, synaesthetic reports of sequences having fixed, object-centered coordinates speaks to that very possibility (Smilek et al., 2007; Eagleman, 2009). As L once described, “When I hear the month January it sits to my right, but then if I see the word January it is all of a sudden now to my left. Even within my spatial maps, I can choose to take on a variety of perspectives by zooming in and out voluntarily.” Being able to move about in one's spatial representation suggests that the spatial forms are not necessarily tied to the synaesthete's body (ego-centric), but rather encompass a coordinate system similar to that of objects (object-centered; Eagleman, 2009). If the ability to change perspectives is a consistent quality of SSS, then it must be incorporated in current theories. To date this characteristic has been described as a mere curiosity (Eagleman, 2009).

Numerous subjective reports from sequence-space synaesthetes have suggested that their fixed spatial representations can be viewpoint variant (Galton, 1880; Seron et al., 1992; Cytowic and Eagleman, 2009). One of the challenges to objectively verifying these subjective claims has been in designing a reliable experimental paradigm to measure the changes in viewpoint. The majority of month-space synaesthetes that we have interviewed report altering their vantage point as the months progress over time or take on the viewpoint of the month cued. If cued with the month April, for example, the vantage point could change for some synaesthetes and appear as if they were all of a sudden standing in front of April. These constant and fleeting changes in viewpoint make it very difficult, not only to document consistency of their month-space, but to capture a reliable change in perspective. For instance synaesthete H reported, “As I moved through the year, I am very aware of my place in the oval at the current time, and the direction I am moving in” (Mann et al., 2009).

Synaesthete L, on the other hand, has two perspectives from which she views her month-space, triggered involuntarily by whether she *hears* or *sees* the month in question (Jarick et al., 2009a, 2011a). In other words, L's perspectives are not dependent on the current month, cued month, or current season. We can reliably trigger changes in L's vantage points by modifying the sensory presentation of the cued stimulus. Empirical confirmation of L's ability to change perspectives has already been established in a previous study (Jarick et al., 2009a), where we have demonstrated opposite response patterns to months and hours depending on the modality of the inducing cue used in a spatial cueing paradigm. For instance, if the word January was presented as a central cue, L responded faster to detect targets on the left because that it was congruent with where January was spatially located (according to her visual spatial map). However, if L was then presented with a voice saying January out loud, she was faster to detect a target on the (opposite) right side because it was now congruent with where January was spatially located (according to her auditory spatial map). This three-way interaction between cue modality (auditory vs. visual), cue type (early vs. late), and target location (left vs. right) was not found for the non-synaesthetes tested. Thus, L's subjective report of being able to change perspectives depending on the modality of the cue was objectively confirmed. In hindsight, these results provided one leg of support for the reification hypothesis in the sense that the month-space is not necessarily tied to the synaesthete (egocentric), but can exists with respect to object-centered coordinates (taking on object-like properties).

**Figure 1:**
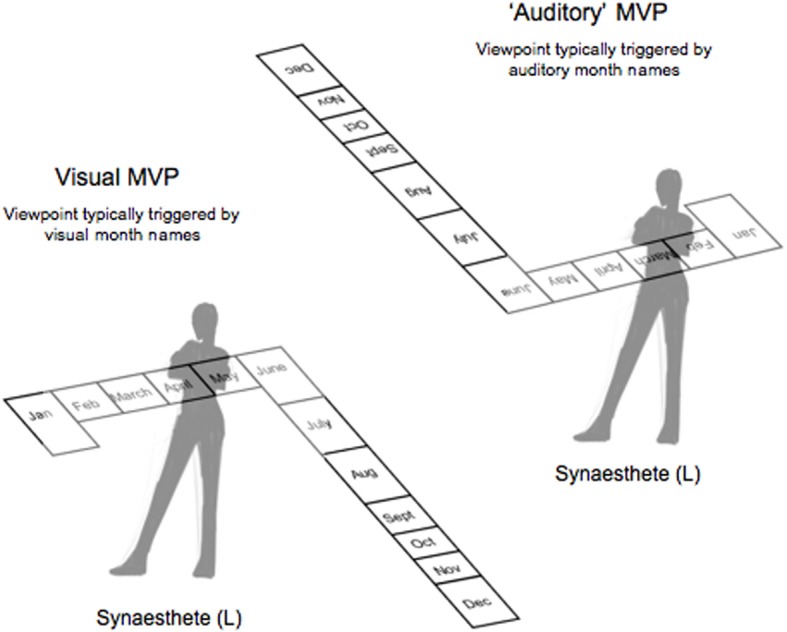
**A schematic depicting the 3D spatial arrangement of L's spatial calendar.** The month cues used in the current experiment were *January, February, March* (early months), and *May, June, July* (late months). The top signifies the mental vantage point (MVP) she takes when the month name is heard (i.e., auditory cue) while the bottom indicates the MVP she takes when the month name is seen (i.e., visual cue).

As we discovered a year or so later, the uniqueness of L's synaesthesia was not limited to her ability to view her time-space from opposite vantage points. L also claimed to prefer to view her space from her auditory viewpoint. As shown in Figure 1, viewing written words from this vantage point causes the words to appear upside-down—a viewpoint for which she would seldom see in real life. By preference, we mean that L views her time-space from the auditory perspective most often and feels most comfortable doing so. Just as one might have a default point of view when we imagine a car (a 3D object that conforms to object-based coordinates), L reports to have a “default” viewpoint of her month-space. As if the month *names* and month spaces encompass objects-based coordinates themselves. According to the reification hypothesis, this would be the case if synaesthetes were treating the months as “objects” and not just a location in space.

In order to test the idea that spatial synaesthetes could have a preferred or “default” mental vantage point for viewing spatial-forms, we focused on the synaesthete L's representation of her months. L has participated in previous studies over the years aimed at verifying her time-spaces (months and hours; Jarick et al., 2009a, 2011a), as well as her number-space (Jarick et al., 2009b, 2011b). As such, the consistency of L's spatial-maps have all been well-established and we have empirically demonstrated that L views her month-space from different perspectives depending on whether the month is *seen* vs. *heard* (Jarick et al., 2009a). In fact, L takes the exact opposite vantage point, making designing an experiment fairly simple. For instance, when L sees a month name written, she views her space organized such that January, February, and March are on her left side and May, June, and July on her right (the rest of the months flow behind her, as seen in Figure 1). When she hears the name of a month spoken, however, she takes the opposite viewpoint and January, February, and March are now on her right side and May, June, and July on her left (the other months extend outward into space). Consistent with the objectification of overlearned sequences (Eagleman, 2009), L reports that she views her month-space from the auditory perspective it is as if she were looking at her months (and month names) literally upside down. What is even more striking about this unusual perspective, is that L claims to *prefer* it that way.

## Experiment 1

To first verify L's claim that her auditory perspective was akin to viewing her months upside down, we tested whether we could elicit her “auditory” vantage point with the *visual* month names alone (i.e., month names written upside down). We used a spatial-cueing task (Posner, 1981) containing two cueing conditions: month names upright or month names inverted. Our predictions were straightforward: we expected that the upright month cues would trigger L's visual perspective and as such, early month cues (*Jan., Feb., March*) should orient her attention to the left side of space (faster to detect left targets), while later month cues (*May, June, July*) should orient her attention to the right (faster to detect right targets). Our key prediction to verify L's claim of viewing months upside down, however, was that the *inverted* month names should trigger the reverse “auditory” perspective. Thus, inverted early months (*Jan., Feb., March*) should orient her attention to the right side of space (faster to detect right targets), while inverted later months (*May, June, July*) should orient her attention to the left (faster to detect left targets), essentially reversing the cueing pattern seen for the upright month cues. If our predictions are confirmed, we should find the same three-way interaction that we previously found (Jarick et al., 2009a,b) between cue condition (upright vs. inverted), cue type (early vs. late), and target location (left vs. right) for L that is absent in non-synaesthetic controls.

### Methods

#### Participants

We tested a 23-year-old time-space synaesthete L and five non-synaesthetic controls from Willamette University, all of which received for a honorarium for their time. All participants had normal or corrected-to-normal vision and hearing and were right-handed. Participants gave informed consent before participating and the board of research ethics at Willamette University and the University of Waterloo approved the experimental procedures.

#### Materials

All stimuli were presented on a white background. Visual month cues were black text (Geneva font, 72 pt). The centrally located cues consisted of 6 month names (height 0.6° visual angle and maximally 6.5° in length)—January, February, March (*early months*), May June, and July (*late months*). Targets were black squares (each side subtending 0.6° of visual angle) and placed at an eccentricity of 10.5° from the center of fixation. Stimuli presentation and data collection were controlled by SuperLab 4.0 experimental software.

#### Procedure

Participants were seated ~57 cm in front of a computer screen and asked to perform a spatial cueing task. A typical trial involved a fixation cross for 600 ms, a central cue (month name) appearing either upright or inverted for 600 ms, followed immediately by a target square to either the left or right of the month cue. The target remained on the screen until the participant responded or 3500 ms elapsed. The task for L and a group of five non-synaesthetic controls was to detect this target as quickly as possible by pressing a central button on a response pad with their dominant (right) hand. Once a response was given, the next trial began. On about 10% of the trials no target would appear (“catch trials”), for which participants were advised to withhold their response until the next trial. These trials were inserted to make certain participants were staying on task. There were four blocks of 132 trials (60 valid, 60 invalid, 12 catch), for a total of 528 trials. Inverted and upright month cues were randomly intermixed within the blocks. Participants had self-paced breaks between blocks.

It is important to note that although the month names served as spatial cues for L, she was aware that they were not predictive of the target location—targets appeared to both the right and left 50% of the time (i.e., half valid, half invalid). Thus, there was no incentive to even pay attention to the month names while detecting the targets presented.

### Results and discussion

The synaesthete L and non-synaesthetic controls performed perfectly on catch trials (100% accurate). The response times for L and the controls were submitted to a recursive outlier procedure, for which observations greater or less than 2.5 standard deviations were discarded. As a result, only 0.65% of data was removed from L, and an average of 1% from controls.

**Figure 2:**
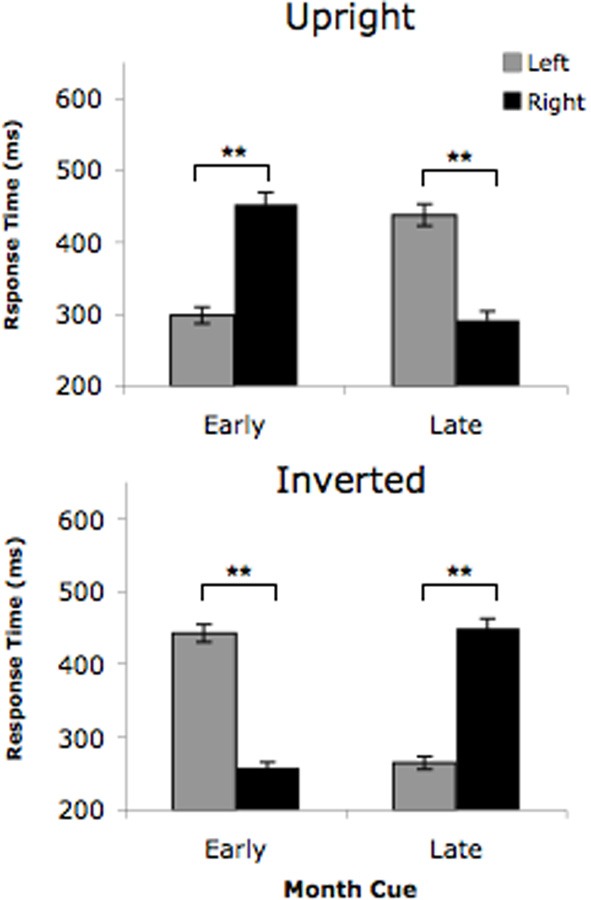
**Time-space synaesthete L's target detection times for the upright month cues and inverted month cues in Experiment 1.** Early month cues consisted of the months *January, February, March*, while late month cues were the months *May, June, July*. Error bars represent the 95% confidence intervals. ^**^p < 0.05.

An analysis of variance (ANOVA) with L's response times showed a significant cue condition (upright vs. inverted) × cue type (early vs. late) × target location (left vs. right) interaction, *F*_(1, 35)_ = 1178.68, *p* < 0.0001, as depicted in Figure 2. That is, when the cue was an upright month name, it elicited L's visual vantage point. As such, she detected targets on the left side faster following early months and detected targets on the right side faster following late months. However, the opposite response pattern resulted when the cues were inverted month names, as they elicited L's “auditory” viewpoint. Thus, following inverted cues, L detected targets on the right faster following early months and faster to detected targets on the left following late months. This three-way interaction suggests that when cued by an early month (for example), L responded significantly faster to targets on the left when the cue was *upright*, but detected targets on the right when the cue was *inverted*. As predicted, these results demonstrate a clear reversal in viewpoint triggered by the two types of visual cues, with the upright cues eliciting the visual vantage point and the inverted cues eliciting the “auditory” vantage point. As expected, not one of the five non-synaesthetic controls showed this three-way interaction (all *p*'s > 0.303). Thus, our findings show that upright and inverted month names cued opposing viewpoints for L, but not for the non-synaesthetic controls. In essence, our data objectively verified L's subjective claim that her “auditory” mental vantage point causes her to view her months upside down. This finding suggests that L's vantage point is not solely dictated by the modality of the inducing stimulus (i.e., a month spoken aloud) like previously believed (Jarick et al., 2009a,b), but rather can be induced by visual stimuli alone as long as the orientation of the stimulus is consistent with L's vantage point of her month-space.

## Experiment 2

Next we tested L's second claim that she *prefers* to view her months from the auditory perspective (i.e., visually upside down). To reiterate, by preference we mean a “default” point of view for which L might show a bias in her behavior toward. To test this, we attempted to create a situation where her visual and auditory viewpoints would be in conflict in order to observe which one she is more biased toward using. If her auditory perspective is indeed her “default” viewpoint, then that perspective should be the viewpoint she takes. Using the same spatial-cueing task, we included four types of cue conditions: auditory-only month names, visual-only month names (upright), audiovisual congruent month names (visual inverted + auditory), and audiovisual *incongruent* month names (visual upright + auditory). Our predictions were as follows: audiovisual congruent cues should not only trigger L's “auditory” perspective, but should facilitate detection of valid targets (due to the multisensory enhancement of having bimodal cues triggering the same perspective). Therefore, the audiovisual congruent cues should produce the largest cueing effects (i.e., difference in response time between valid and invalid targets) compared to auditory-only cues. Our key prediction, however, was regarding the audiovisual incongruent condition. We hypothesized that when the month cues trigger both perspectives simultaneously (i.e., auditory cue triggers “auditory” perspective and visual upright cue triggers visual perspective), the preferred “auditory” perspective should be the one elicited. That is, if L prefers her auditory viewpoint, then she should show a bias in her response pattern consistent with the “auditory” perspective (i.e., similar to the auditory-only condition).

### Methods

#### Participants

The time-space synaesthete L participated for a honorarium. We did not include non-synaesthetic control participants, as this examination was purely a test of L's ability.

#### Materials and procedure

Stimuli were the same as Experiment 1, except the two audiovisual (AV) conditions were added. The AV congruent condition elicited the same vantage point for L and consisted of the visual month name inverted paired simultaneously with the month spoken aloud from the computer loudspeakers (both cues triggered the “auditory” perspective). Both visual and auditory stimuli were similar in the degree of saliency to the participant, in that the month name appeared on the computer screen at a comfortable reading size (height of 0.6° visual angle and maximally 6.5° in length) and the voice was presented at a comfortable hearing level (~65 dB). The AV incongruent condition should elicit conflicting vantage points for L and consisted of the visual month name upright paired simultaneously with the month spoken aloud from the computer loudspeakers (visual cue triggered visual perspective while auditory cue triggered “auditory” perspective). Stimuli were presented in two blocks, each containing 30 valid and 30 invalid of each type of cue (visual-only, auditory-only, AV congruent, AV incongruent) plus 12 catch trials, all randomly intermixed. Participants L had a 5-min break between blocks. There were 528 trials in total and the experimental session lasted about 20 min.

### Results and discussion

L performed perfectly on catch trials (100% accurate). Only 0.01% of L's data was removed by the outlier procedure (±2.5 standard deviations).

**Figure 3:**
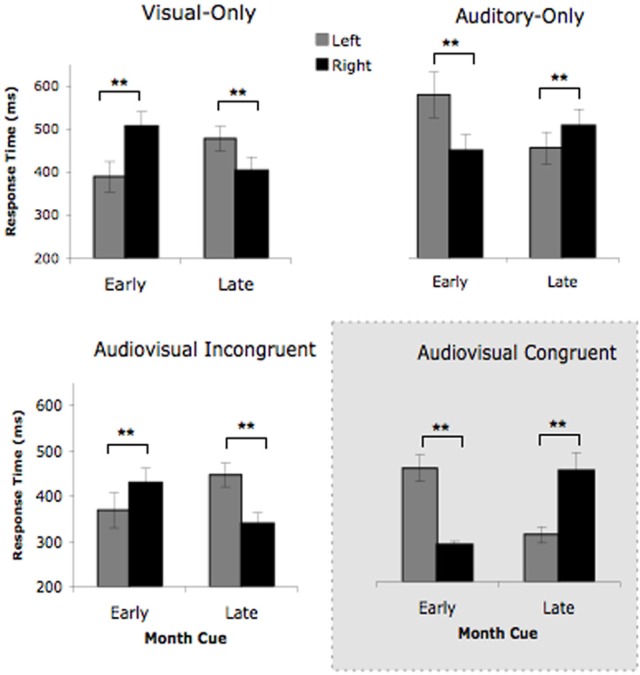
**L's target detection times for the four cue conditions in Experiment 2.** Early month cues consisted of the months *January, February, March*, while late month cues were the months *May, June, July*. The Visual-Only condition contained visual month cues that were upright. The Auditory-Only contained auditory month cues that were spoken aloud over loudspeakers. The Audiovisual Incongruent cues contained auditory month names paired with the visual month name upright (triggering conflicting viewpoints), while the Audiovisual Congruent contained auditory month names paired with the visual month name inverted (triggering the same viewpoint). Error bars represent the 95% confidence intervals. ^**^p < 0.05.

To evaluate which viewpoint each cue condition would elicit (i.e., whether L's response pattern would be biased toward her “auditory” or visual perspective), we conducted a 4 × 2 × 2 repeated measured ANOVA with the factors cue condition (auditory-only, visual-only, AV incongruent, and AV congruent), cue type (early and late), and target location (left and right). The ANOVA revealed a significant main effect of cue condition, *F*_(2.28, 72)_ = 30.97, *p* < 0.001, and a significant two-way interaction between cue type and target, *F*_(1, 24)_ = 6.27, *p* < 0.05. The key finding was the significant three-way interaction between cue condition, cue type, and target location, *F*_(3, 72)_ = 46.36, *p* < 0.001, which suggested that the different cue conditions were eliciting different vantage points for L. The results can be seen in Figure 3.

To investigate this three-way interaction further, we conducted a 2 (cue condition) × 2 (cue type) × 2 (target location) ANOVA for each unimodal (auditory-only vs. visual-only) and bimodal (AV congruent vs. AV incongruent) conditions separately. The unimodal conditions have been tested previously (Jarick et al., 2009a), for which a significant three-way interaction was found that depicted the vantage point reversal between visual and auditory cues. In other words, the auditory cues elicited the “auditory” viewpoint and the visual cues elicited the opposing visual viewpoint. Based on our previous findings, we predict the same three-way interaction for the unimodal conditions here. Not surprisingly, our prediction was confirmed and the ANOVA revealed a significant three-way interaction between cue condition, cue type, and target location, *F*_(1, 27)_ = 34.93, *p* < 0.001, again replicating the opposing viewpoints triggered by the visual-only and auditory-only cues. The ANOVA also showed significant main effects of cue condition, *F*_(1, 27)_ = 19.93, *p* < 0.001, and cue type, *F*_(1, 27)_ = 4.71, *p* < 0.05, as well as a significant two-way interaction between cue condition and target, *F*_(1, 27)_ = 6.24, *p* < 0.05. No other comparisons were significant. Therefore, consistent with previous findings (Jarick et al., 2009a,b), L responded in a manner consistent with her visual perspective when cued by the visual-only upright months, as well as the “auditory” perspective when cued by the auditory-only months.

The more interesting finding is whether L showed a bias toward her preferred “auditory” viewpoint. This was tested in the AV incongruent condition, where the cues triggered conflicting vantage points. Thus, our predictions for the bimodal conditions really depend on which vantage points L is biased toward in the AV incongruent cue condition. We predicted that the AV congruent cues (auditory + month inverted) would undoubtedly trigger L's “auditory” vantage point, since we have already shown that each does individually from the unimodal analysis. The AV incongruent cues (auditory + month upright), on the other hand, were expected to trigger both visual and “auditory” perspectives, thereby putting L in conflict. Due to L's declared preference for her auditory perspective, we predicted that the “auditory” viewpoint would be more strongly elicited. If this is the case, we should not find a significant three-way interaction due to both cue conditions showing similar cueing patterns, but rather a two-way interaction between cue type and target location. Contrary to our predictions, the ANOVA did reveal a significant three-way interaction between cue condition, cue type, and target location, *F*_(1, 26)_ = 155.27, *p* < 0.001, which indicated that the AV incongruent cues elicited L's visual vantage point. Visual inspection of Figure 3 illustrates the perspective reversal (i.e., three-way interaction), such that the AV incongruent cues (i.e., when the cues were in conflict) elicited the same mental vantage point as the visual-only cues, while the AV congruent cues (when the cues were complementary) elicited the same mental vantage point as the auditory-only cues.

At first glance, this latter finding seems to falsify L's claim of preferring the “auditory” viewpoint, since when pitted against one another, the visual viewpoint was the one that biased L's attention. However, there are other possibilities to consider. Perhaps when the cues triggered opposite vantage points, L needed to reconcile the conflict with a strategy. Intuitively speaking, the simplest strategy in this case would be to actively ignore one of the cues and in this situation (i.e., being visual detection task) the auditory cue is easiest to suppress. In other words, it would be more difficult to suppress visual information and simultaneously perform a visual task. It would therefore be easiest for L to suppress the auditory information and focus on the visual, which would result in response times consistent with her visual perspective. Another possibility is that even though L has a claimed preference for the “auditory” perspective this does not mean that the auditory perspective will be dominant. It is well-known that vision is the dominant sense, and it would not be too far fetched to believe that the visual cues would be dominant in determining L's vantage points.

**Figure 4:**
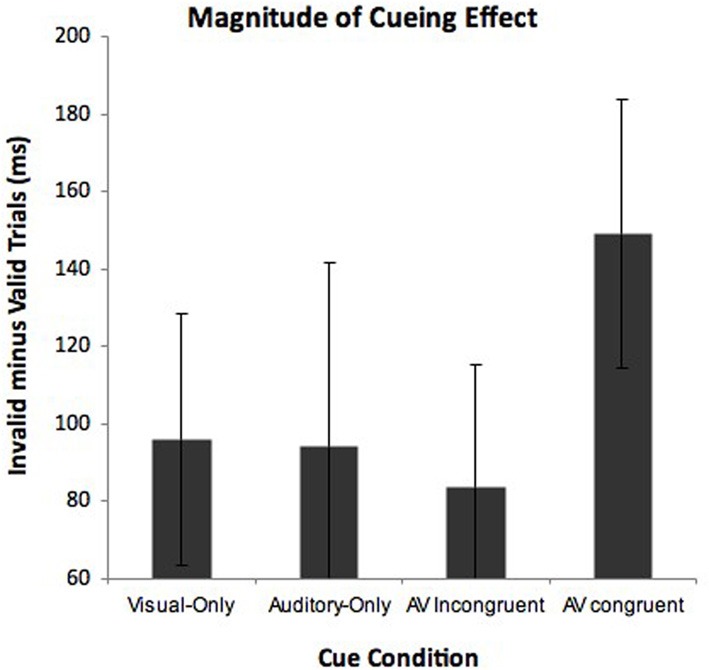
**Magnitude of L's cueing effects for each cue condition in Experiment 2.** Cueing effects were calculated by subtracting response times to detect valid targets (target appeared at cued location) from response times to detect invalid targets (target appears in uncued location). Error bars represent 95% confidence intervals.

There is, however, another avenue to evaluate L's claim of her preferred “auditory” vantage point—to analyze the *magnitude* of the cueing effects (i.e., response time difference between valid and invalid targets) within the cue conditions. To get a measure of magnitude, we calculated response times to detect valid targets (target in cued location) and invalid targets (target in uncued location) for each of the cue conditions. We performed a repeated measures two-way ANOVA with the factors cue condition (visual-only, auditory-only, AV congruent, and AV incongruent) and validity (valid vs. invalidly cued targets). Notably, the “valid” trials in the AV incongruent condition were with reference to the vantage point L took that being the visual perspective. We predicted that due to the multisensory nature of the AV conditions, both would show a multisensory benefit with larger cueing effects compared to the visual-only or auditory-only conditions. In terms of L's preference for her auditory viewpoint, the AV congruent cues should show a significantly stronger cueing effect compared to the other cue conditions. The ANOVA showed a significant main effect of cue condition, *F*_(3, 177)_ = 38.97, *p* < 0.001, and a main effect of validity, *F*_(1, 59)_ = 104.28, *p* < 0.001. Cue condition and validity was also shown to interact, *F*_(3, 177)_ = 2.58, *p* = 0.05. To interpret this interaction we calculated cueing magnitude scores (invalid response times minus valid response times). These cueing magnitude scores are shown in Figure 4. *Post-hoc* (LSD) comparisons indicated that cueing magnitudes were greatest in the AV congruent condition than the visual-only (*p* < 0.05), auditory-only (*p* < 0.05), and AV incongruent condition (*p* < 0.05). The visual-only, auditory-only and AV incongruent conditions did not differ at all (all *F*'s < 1 and *p*'s > 0.05). Although the audiovisual conditions both showed faster overall response times compared to the unimodal conditions, only the AV congruent cues differed in the magnitude of their cueing effects. Specifically, following the AV congruent cues (both elicited auditory vantage point), L showed the greatest cost when the target was invalid, but also the greatest benefit when the target was valid. This finding is consistent with L's claim of preferring the viewpoint elicited by the AV congruent cues (i.e., “auditory viewpoint”).

## Conclusions

Together our data show that L's performance in the spatial-cueing task was clearly influenced by her extraordinary month-space associations in which the vantage point from where she views her month-space has a profound influence on her spatial attention. In the first experiment we were able to capture L's auditory mental vantage point MVP using *visual* stimuli alone and in the second experiment show some objective support for her claim that she prefers to view her space from the “auditory” perspective. Thus, far, L is the only synaesthete who has shown these unique traits. Other accounts of time-space synaesthesia have reported being able to “zoom in and out” of their spatial forms or report feeling like they are walking along with the months as they pass through the year (Galton, 1880; Seron et al., 1992; Cytowic and Eagleman, 2009), but none who report reversing their perspective with reference to an inducing stimulus.

The finding that L can reverse her perspective on “time” is not new (Jarick et al., 2009a,b), however our finding that she vividly experienced the month names upside down when viewing her month-space from the “auditory” perspective is very informative. It perhaps suggests that her month-space does not always adhere to egocentric coordinates and could exhibit object-like properties. The fact that we could induce both of her vantage points using visual stimuli alone speaks to how visually detailed L's month-space is for her. Just as we can be influenced to view objects from different perspectives, L too can view her spatial calendar from different mental vantage points. Her report of having a preferred viewpoint is also consistent to how we might imagine objects from a canonical perspective. Keeping in mind of course that our mental viewpoints are initially the product of viewing real objects in the external world, while L's viewpoints are completely internally generated. This evidence can provide some behavioral support for Eagleman's *reification hypothesis*. Of course this is a first step and only one synaesthete; more data is needed to generalize these findings to other synaesthetes.

However, if it is the case that these implicit sequences trigger an experience of objecthood for synaesthetes, theories regarding the development and neural architecture of synaesthesia need to take that characteristic into account. For instance, previous studies (Hubbard et al., 2005; Tang et al., 2008) have primarily focused on the parietal lobes to uncover the neural correlates of SSS, being that the seeing as though the parietal areas process visuo-spatial information including well-learned sequences. However, as Eagleman (2009) suggests, perhaps we should also be looking at activity in the temporal lobes where properties of objects are represented. In fact, Pariyadath et al. (2008) have recently shown brain activation in temporal lobe areas during the processing of overlearned sequences in non-synaesthetes. Temporal lobe activation for SSS has yet to be confirmed, however it would provide some clues as to why many sequence-space synaesthetes also see colors for the months of the year and days of the week (Sagiv et al., 2006; Smilek et al., 2007).

What is further unique about L is that she possesses a strong preferred viewpoint that is unconventional to what most people would prefer, what most people would be “taught.” She prefers to view her space upsidedown, where the average person would intuitively feel more comfortable viewing months written upright. In terms of the developmental debate of synaesthesia, some argue that it is simply the product of learning. However, if that were the case, it is unclear what might have motivated L to learn a mental calendar that would be upsidedown.

### Conflict of interest statement

The authors declare that the research was conducted in the absence of any commercial or financial relationships that could be construed as a potential conflict of interest.
